# L-band radar quantifies major disturbance of birds by fireworks in an urban area

**DOI:** 10.1038/s41598-023-39223-1

**Published:** 2023-07-26

**Authors:** Joseph P. Wayman, George Atkinson, Mohammed Jahangir, Daniel White, Thomas J. Matthews, Michail Antoniou, S. James Reynolds, Jon P. Sadler

**Affiliations:** 1 School of Geography, Earth and Environmental Sciences, University of Birmingham, Birmingham, Edgbaston, UK; 2 Microwave Integrated Systems Laboratory, School of Engineering, University of Birmingham, Birmingham, Edgbaston, UK; 3 CE3C – Centre for Ecology, Evolution and Environmental Changes/Azorean Biodiversity Group/CHANGE – Global Change and Sustainability Institute and Universidade dos Açores – Faculty of Agricultural Sciences and Environment, PT-9700042, Angra do Heroísmo, Açores Portugal; 4 Birmingham Institute of Forest Research, University of Birmingham, Birmingham, UK; 5 Centre for Ornithology, School of Biosciences, College of Life and Environmental Sciences, University of Birmingham, Edgbaston, Birmingham, UK; 6 The Army Ornithological Society (AOS), c/o Prince Consort Library, Knollys Road, Aldershot, Hampshire UK

**Keywords:** Urban ecology, Urban ecology

## Abstract

Fireworks and other pyrotechnics are acknowledged as sources of disturbance to wildlife, with evidence that many species react adversely to their sight and sound at discharge. However, how firework releases impact wildlife within a city landscape is poorly understood. Here, we explore the effect of fireworks on urban birds using an L-band staring radar (90-degree sector out to a 5 km range) to capture bird activity derived from flight tracks (i.e. 3D visualisation of individual flying birds built from radar detections) within the city of Birmingham, UK. Comparing the tracks between baseline periods with no fireworks and periods where fireworks are commonly discharged using a null model indicated that birds flew at higher elevations during firework periods (standardised effect sizes of 17.11, 26.54 and 5.83, for Diwali, Bonfire Night, and New Year's Eve, respectively). Birds also flew in more significant numbers (standardised effect sizes of 23.41, 7.98 and 7.19 for Diwali, Bonfire Night, and New Year's Eve, respectively). Therefore, bird activity was elevated during firework events at a time of night when many would otherwise be roosting. Such disturbance may have implications for avian biology since large public firework events occur at colder times of the year in the UK when birds have elevated thermoregulatory costs.

## Introduction

Wildlife has long been known to experience various impacts from anthropogenic disturbance including noise (e.g., from motor vehicles, planes, construction, alarms etc.), recreation (e.g., hunting, ecotourism), and energy generation and infrastructure (e.g., wind farms, oil and gas extraction)^1–7^. Noise from such disturbance sources can disrupt the behaviour of terrestrial and marine wildlife (see Shannon et al.^6^ and Bowles^8^ for reviews) and cause adverse physiological reactions^6,9–11^. Firework displays are sporadic sources of anthropogenic disturbance that negatively impact acoustic and visual modalities of wildlife^12,13^. Meanwhile, the use of pyrotechnics as a deterrent to disperse birds at airports or on crops suggests that birds are susceptible to such disturbances^14–16^. Stickroth^17^ reviewed avian disturbance from fireworks and found 133 observations of 88 species of bird responding to visual and auditory stimuli from fireworks, resulting in nest abandonment, panic flights and temporary exclusion from areas close to the firework discharge. Fireworks also affect birds physiologically by increasing their heart rates and cortisol production^11,18^. Whilst fireworks are released during daylight hours for various reasons, such as concerts and as a deterrent to disperse birds^14–16^, they are most often released after sunset to maximise the light display. Therefore, the lack of studies linking avian activity to firework releases may in part be due to birds being challenging to track in large numbers as well as the difficulty in making contemporaneous observations of them during hours after sunset when fireworks are typically discharged.

Another research gap in relation to the impact of fireworks on birds is the effect of fireworks on birds inhabiting large urban areas. In his review, Stickroth^17^ showed that across studies, fireworks impacted birds more in open country than in woodlands. However, the exact cause of this difference in disturbance, postulated as a difference in species' sensitivity, increased predation threat (if fireworks are perceived in this way by birds), or an increase in the intensity of fireworks over the open country, is unknown. As highly urbanised areas (such as cities) are a specific type of closed habitat with largely impervious surfaces and species assemblages composed primarily of generalists that are disturbance-habituated^19^, the response within cities may be different from responses previously observed in non-urban environments. These generalist species, many of which are truly cosmopolitan species^20^, such as feral pigeons (*Columba livia domestica*) and house sparrows (*Passer domesticus*), often thrive in human-dominated landscapes and they may have become largely habituated to loud, frequent anthropogenic noise sources.

**Figure 1:**
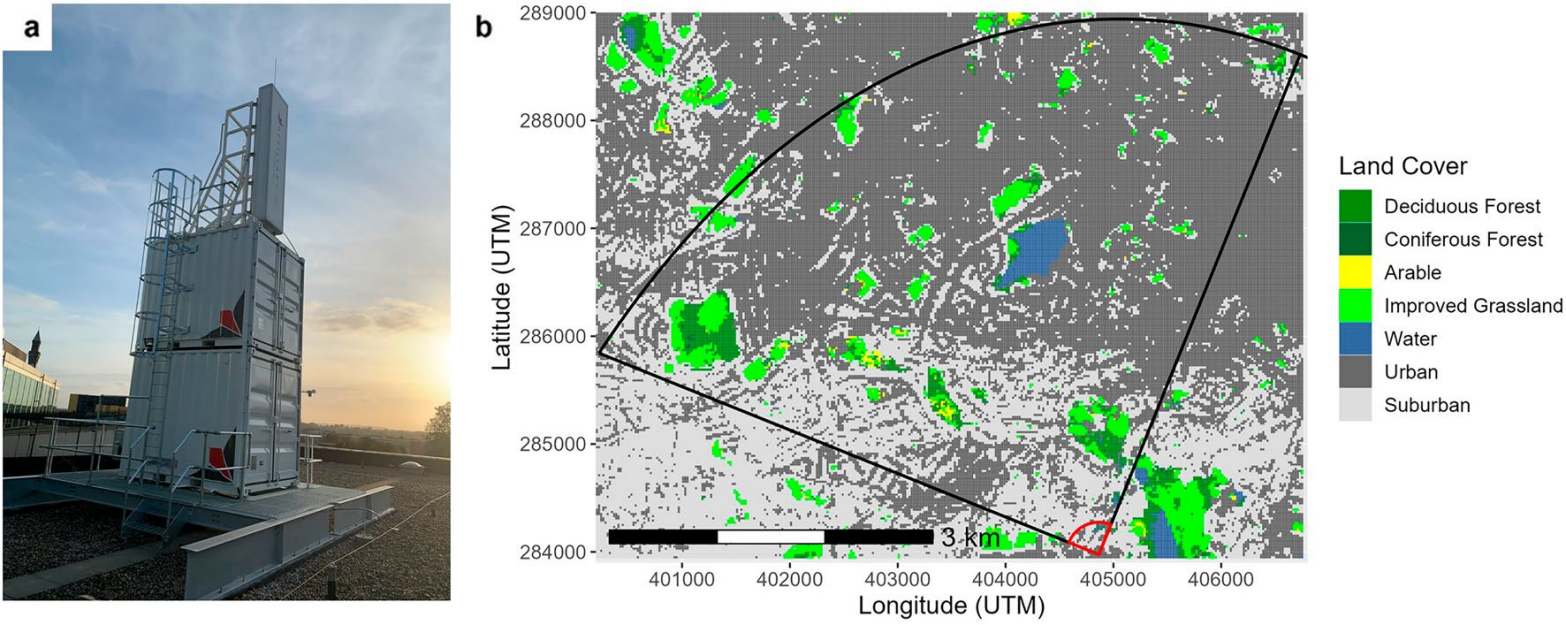
(**a**) The Thales Avelliant L-band prototype staring radar used in the study. (**b**) The study area, as defined by the black-lined segment representing the radar’s field of view (a 90-degree azimuth sector out to a 5 km range), overlooking the city of Birmingham, UK. The city is mainly composed of urban and suburban land cover. The red-lined segment was not monitored due to its proximity to the radar. Data to produce the map were obtained from: Land Cover map of Great Britain (2019) [TIFF geospatial data], Scale 1:250,000, Tiles: GB, Updated: 30 June 2020, CEH, Using: EDINA Environment Digimap Service, https://digimap.edina.ac.uk, Downloaded: 2021-05-05 09:48:48.703.

Here, using data generated from an L-band staring radar (Fig. 1a), we provide an assessment of the impact of fireworks during three time periods when fireworks are commonly released (Diwali, Bonfire Night (BN), and New Year’s Eve (NYE)) on bird populations within Birmingham, UK (the UK's second-largest city; Fig. 1b) during 2021. While weather radars, more commonly used for ecological studies, allow for large-scale (~ 7800 km^2^) disturbance of bird populations to be quantified^21,22^, staring radar, which covers a relatively smaller area (20 km^2^), permits tracking of individual birds^23^, allowing for finer scale measurement, albeit at a smaller spatial scale. Furthermore, staring radars can operate at lower altitudes in urban areas where ground clutter (from the constructed landscape) can impede data capture from other systems^21^. In this study, we compared the number and heights of radar tracks (i.e., multiple detections of a single target, here used as a proxy for bird flighted activity) measured during the release of fireworks with those from baseline periods (one for NYE and one for BN and Diwali) that were temporally close to discharge dates. Firework release times were selected based on knowledge of the events and air pollution data. We hypothesised that birds (i.e., radar tracks of flighted bird targets) will be more numerous and recorded at higher altitudes during periods of firework release (following Kölzsch et al.^11^ and Shamoun-Baranes et al.^21^) as birds disperse from the disturbance source, potentially flying higher as an escape response to the noise and/or illumination generated from the fireworks. Alternatively, there may be no difference in the number of tracks or the height of those tracks between the baselines (when no or few fireworks are discharged) and firework event times due to the landscape or the composition of the bird community, based on the aforementioned differences.

## Results

### Flight Heights

**Figure 2:**
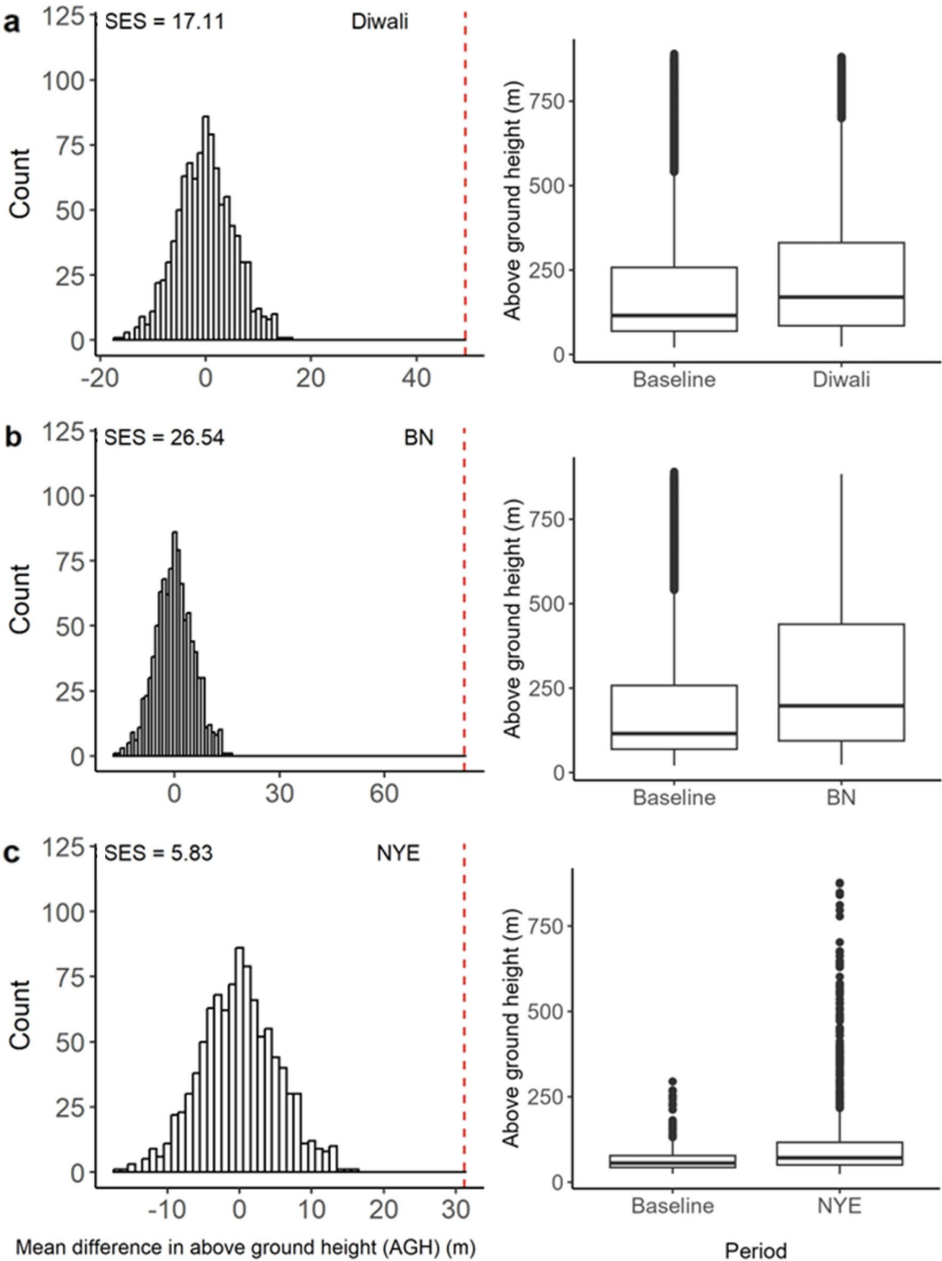
(left) Null distributions of the mean difference in average above ground height of targets recorded on the radar between the firework events on (**a**) Diwali, (**b**) Bonfire Night (BN) and (**c**) New Year’s Eve (NYE) and the relevant baseline period. The red vertical dashed lines show the observed mean difference between each firework event and the relevant baseline period. Standardised effect sizes (SES) are displayed in the top left corners. On the right of each event (**a–c**) is a boxplot showing the median (box midline), the inter-quartile range (the box), and 1.5 times the inter quartile range (the whiskers) for each firework event and the respective baseline period. Points outside of the whiskers represent outliers.

Recorded radar tracks (a proxy for flighted activity of birds, with a single track constituting continued observation of one or more birds in flight) during Diwali (261 ± 222 m standard deviation) and BN celebrations (283 ± 229 m) were recorded at higher average altitudes than during the corresponding baseline periods (200 ± 194 m) (Fig. 2a,b). Tracks were also higher during NYE, although their average above ground heights (AGHs) were lower during firework release (116 ± 127 m) and the baseline period (85 ± 79 m) than during the Diwali and BN periods and baseline periods (Fig. 2c). When comparing the observed mean difference to a calculated null distribution, tracks during Diwali (standardised effect size (SES) = 17.11, *p* < 0.001; Fig. 2a), BN (SES = 26.54, *p* < 0.001; Fig. 2b), and NYE (SES = 5.84, *p* < 0.001; Fig. 2c) were all at significantly higher altitudes during firework events than during corresponding baseline periods. The average minimum track height during Diwali (224 ± 210 m, SES = 16.26, *p* < 0.001), BN (256 ± 225 m, SES = 25.82, *p* < 0.001), and NYE (96 ± 121 m, SES = 5.18, *p* < 0.001) were all significantly higher than during their respective baseline periods (178 ± 189 m for Diwali and BN and 70 ± 73 m for NYE). The same was true for the average maximum height during Diwali (277 ± 223 m, SES = 17.79, *p* < 0.001), BN (311 ± 236 m, SES = 27.10, *p* < 0.001) and NYE (138 ± 135 m, SES = 6.22, *p* < 0.001), with all significantly higher than during their respective baseline periods (224 ± 201 m and 102 ± 89 m for Diwali and BN, and 102 ± 89 m for NYE).

### Activity

**Figure 3:**
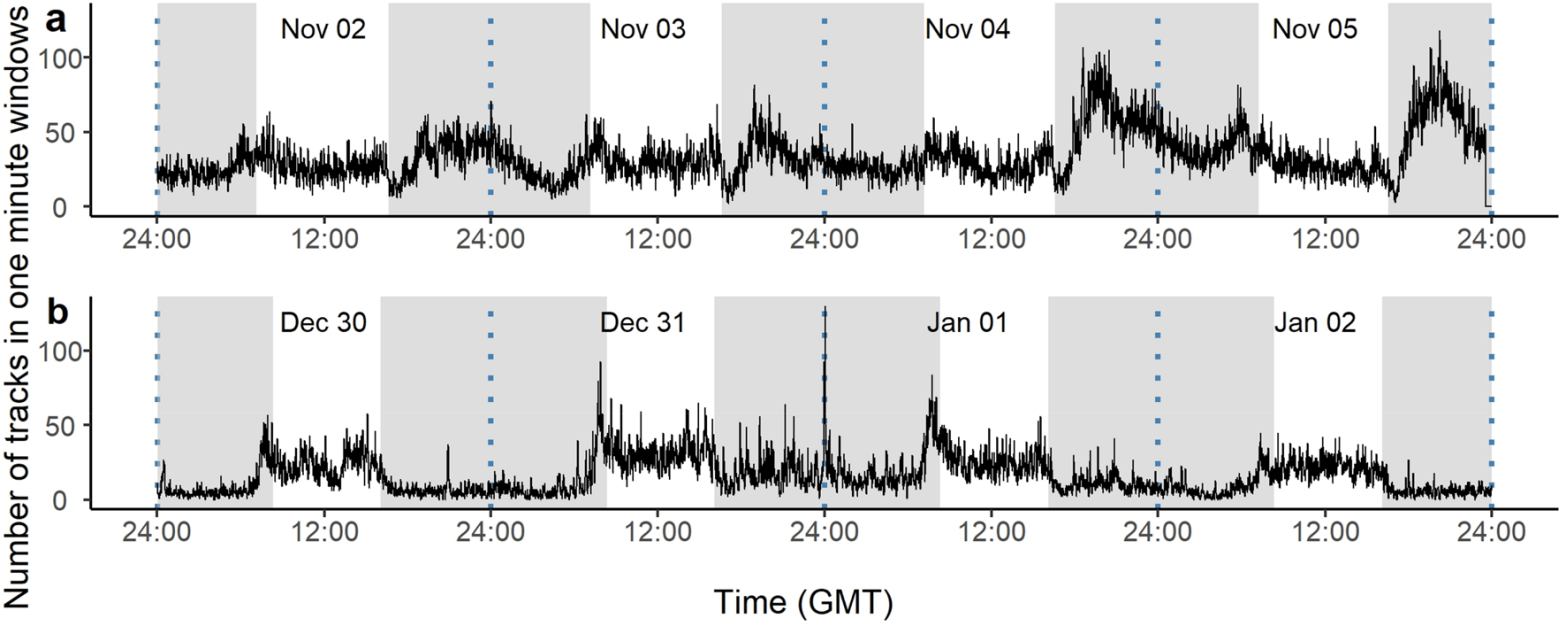
The number of tracks of birds in one-minute rolling windows within the radar's field of view during firework events during (**a**) Diwali (04/11/21), and Bonfire Night (05/11/21; BN), and (**b**) New Year's Eve (midnight on 31/12/21; NYE) compared with baseline periods of 10/29/21–03/11/21 for Diwali and BN, and 28/12/21–03/01/22 for NYE in Birmingham, UK. Vertical dashed lines show midnight, and grey-shaded areas show periods between sunset and sunrise each day.

Using one-minute rolling intervals, there were discrete peaks in the number of recorded radar tracks aloft during periods of firework discharge compared with baseline periods for each event (Fig. 3). The total number of tracks within a one-minute window peaked at 105 (20.07 h), 118 (20.13 h), and 132 (00.02 h) through firework releases during Diwali, BN and NYE celebrations, respectively. Activity peaks were coincident with the peaks in the air pollution data, which correlated with the periods when firework activity was expected (i.e., after sunset) during Diwali and BN (supplementary Fig. S1.1) and just after midnight on 31/12/2021 on NYE (Fig. 3b).

**Figure 4:**
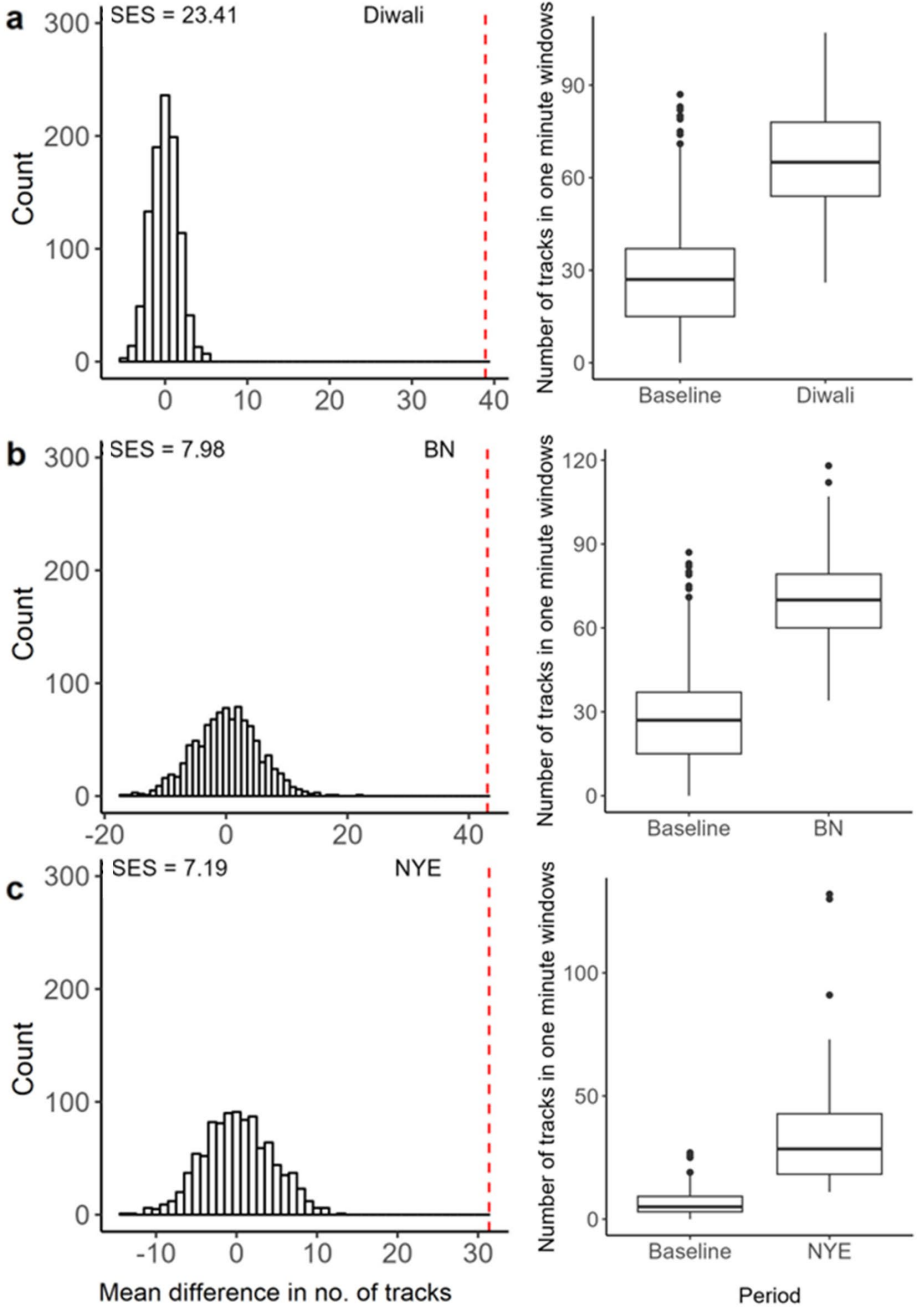
(left) Null distributions of the mean difference between the firework events on (**a**) Diwali, (**b**) Bonfire Night (BN) and (**c**) New Year’s Eve (NYE) and the relevant baseline period for the number of tracks recorded on the radar binned into one-minute windows. The red vertical dashed lines show the observed mean difference between each firework event and the relevant baseline period. Standardised effect sizes are displayed in the top left corners. On the right of each event (**a–c**) is a boxplot showing the median (box midline), the inter-quartile range (the box), and 1.5 times the inter quartile range (the whiskers) for each firework event and the respective baseline period. Points outside of the whiskers represent outliers.

The total number of recorded radar tracks (activity) was significantly greater during firework events than during baseline periods. The total numbers of tracks across the 4-h period (i.e. between 18.00 and 22.00 h) for Diwali (16,874) and BN (15,883) were larger than the same temporal windows of baseline periods (9,055 ± 3,560). Within the one-minute intervals, Diwali (66.2 ± 17.0) and BN (70.3 ± 13.8) had, on average, more tracks than the baseline (27.2 ± 14.6) (Fig. 4a,b). Compared to a null distribution of mean differences (i.e., the difference in the mean altitude obtained from two sets of data randomly sampled from the joint firework and baseline data), Diwali (SES = 23.41; Fig. 4a) and BN (SES = 7.98; Fig. 4b) had significantly more tracks. Both the total number of tracks (1,146) and the average number of tracks within each one-minute interval (38.2 ± 31.2) between 00.00 and 00.30 h were also greater during NYE than during the baseline period (i.e. 350.0 ± 99.0 and 6.7 ± 5.7, respectively), representing a significant difference (SES = 7.19 for average number of tracks; Fig. 4c).

## Discussion

Birds respond behaviourally to noise and other types of disturbance from a broad spectrum of anthropogenic sources, including fireworks^6,17^. Here, using an L-band radar overlooking a large conurbation in central England, we show that birds within an urban landscape respond to fireworks by taking to the skies when the majority of diurnal bird species within the area would otherwise be sleeping. This finding confirms and extends the results of previous studies^21^ by providing evidence that, similar to birds in rural landscapes, those occupying urban localities take to the skies in greater numbers (as highlighted by the increased number of recorded tracks) and fly at higher altitudes upon the release of fireworks. 

### Activity levels 

The current research addressing the impacts of fireworks on birds has focussed on rural or natural (i.e., non-urban) areas and primarily on waterfowl^17,21,24,25^. Evidence suggests that they (waterfowl), and other flocking birds (such as common starlings (*Sturnus vulgaris*)), are particularly prone to disturbance from fireworks. Bird species that forage and roost in flocks within urban areas (e.g., feral pigeons and house sparrows) are also, therefore, likely to be commonly disturbed by fireworks released in urban areas. Indeed, research has shown that the reproductive output of house sparrows drops when exposed to firework disturbance in Spain^26^. Furthermore, water bodies often exist in close proximity to urban areas, such as Edgbaston Reservoir in our study area (Fig. 1b). So, waterfowl may constitute many of the tracks we observed. However, further research is needed to confirm pyrotechnics as a significant source of anthropogenic disturbance for individual species within urban confines.

The numbers of night-time tracks during the baseline period temporally close to Diwali and BN celebrations were high compared to the baseline period of NYE. Although this increased activity could indicate fireworks outside of Diwali and BN, it is more likely this was due to migration activity. Indeed, nocturnal migration activity of birds, such as thrushes (Turdidae), could have occurred during the recording period for BN and Diwali as migration still occurs into mid-November^27^. However, migration activity would have had to peak during firework events on BN and Diwali and not be present during the selected corresponding baselines to cause significant differences between those nights and those in the baselines. Information from a Trektellen station positioned at Monkspath, Solihull (~ 12 km south-east of our radar's location) on nocturnal migration flight calls shows that activity for the most common migrant, namely redwing (*Turdus iliacus*), peaked on 12/10/2021 at 2,811 individuals (supporting Fig. S1.2). This migration activity resulted in a large number of recorded radar tracks on the radar (a proxy for activity) across that evening, but the numerical peak and total activity were lower than during Diwali and BN. Unfortunately, there were no data for migrants from the Monkspath station for the firework events in our study, perhaps due to the difficulty in recording flight calls during firework release. Therefore, migrant activity may be an important consideration for future studies researching firework disturbance during periods of migration activity. However, it is important to note that more work needs to be conducted to identify the drivers of increased activity across the selected baseline period as other factors could also influence the number of recorded tracks (e.g., weather and avian population density). 

The number of tracks (activity) around midnight on NYE is strong evidence of birds taking to the skies in response to fireworks, given this is outside the main passage migration window. The recorded number of tracks during NYE was also much greater than that observed during the daily dawn and dusk peaks when birds are generally more active^28^. Not only did the number of tracks increase during firework discharge (Figs. 2 & 3), birds also appeared to fly higher than during baseline periods (Fig. 2). 

The increased activity around firework releases concurs with Shamoun-Baranes et al.^21^, who found that the number of birds aloft detected with weather radar in The Netherlands increased along with their altitude (> 500 m) in response to firework discharges during NYE celebrations. However, fundamental differences between our UK study and this Dutch study in terms of duration of disturbance (~ 5 *versus* 30 min), habitat type (urban *versus* natural open), monitoring hardware (L-band *versus* weather radar) and avian community structure make direct comparison problematic. In addition, public firework displays in the UK during our study period were severely truncated or even cancelled due to the COVID-19 pandemic, especially at the time of NYE in 2021 when the Omicron variant was spreading in the UK. The increased restrictions meant that the number of release sites, and therefore fireworks released, within Birmingham during NYE were probably reduced compared with those during Diwali and BN, and certainly to a greater extent compared with the Dutch study^21^. Firework release across The Netherlands is also usually very high on NYE, with PM_10_ concentrations (particulate matter 10 microns or less) increasing tenfold on average in the first hour after midnight compared to average concentrations outside NYE^29^. However, despite all of the highlighted differences between the two studies, the response of the birds to fireworks across the two are categorically aligned. Whilst this highlights a potentially pervasive impact of fireworks on birds, it also shows the potential of radar systems such as the one used here for longer term monitoring of flighted activity to capture responses to events such as firework displays. 

When comparing the number of tracks on NYE with those during Diwali and BN, UK restrictions on public events as a result of COVID-19 may have meant that disturbance was greater during the latter than during the former (as there were no cancellations of large events during Diwali or BN, but individual displays (e.g., firework releases from private gardens), could have been impacted). Furthermore, reduced avian activity on NYE could have resulted from concentrated fireworks around midnight compared to their more protracted discharges during Diwali and BN^30^ (Supplementary S1.1). Therefore, variability in the intensity, locations and timings of firework releases between the three focal celebrations may have created an environment of disturbance, causing birds to be ‘chased back and forth’ by releases from differing locations and times^17^. Alternatively, the timings of the events (November (Diwali and BN) *versus* late December (NYE)) could have produced the differences observed in activity levels due to contrasts in migration activity or perhaps due to the number of individual birds within the city during those periods. However, more data need to be collected over a longer timeframe during periods of firework release to confirm what factors are driving the differences in the responses. 

### Potential impacts of fireworks on birds

Birds respond behaviourally and physiologically to sounds ≥ 40 dBA (A-weighted decibels, noise adjusted relative to frequency)^6,31^. For example, great tits (*Parus major*) increased vigilance during aircraft flyovers, reducing their feeding efficiency and, ultimately, their fitness^31^. Compared to this noise level, fireworks have a legislated upper noise limit of 120 dBA, equivalent to thunderclaps. However, in contrast to fireworks, birds react to thunderstorms early due to environmental cues such as increases in wind speed and reductions in air pressure in association with an approaching storm^32–34^. There is no similar environmental pathway through which birds can anticipate an impending firework release. Firework releases are also temporally unpredictable to birds, increasing the avian stress response^35^ and negating habituation. Thus, even after previous exposure to fireworks, birds will likely remain within the release area and only flee once the disturbance starts^17^. However, studies have shown that certain long-lived bird species, such as mallards (*Anas platyrhynchos*)^24^, pink-footed geese (*Anser brachyrhynchus*)^11^, and birds of prey (that may not perceive fireworks as a threat, although this may not be the case in British birds of prey due to past persecution^36^) are impacted to a lesser degree than other species such as passerines (that are fast-living species and, therefore, may have a greater emergency response)^17,37–39^. There is some evidence of repeated disturbance within our study area. As seen from the increasing pollution (see supplementary Fig. S1.1) associated positively with firework releases, bird activity continued to increase during the evenings of Diwali and BN. Thus, birds within the area may have been taking to the air in repeated flights, or, alternatively, they were increasingly displaced from surrounding areas and crossed the radar coverage area. However, more work, potentially using GPS-tagged individuals, must be completed to confirm whether birds were taking to the air in repeated flights with each firework release.

Whilst direct mortality from fireworks is unlikely (*cf*. Stickroth^17^), the disorientation caused to volant birds by the sudden noise, light, and other visual cues (such as the confetti released from some fireworks) from a released firework may increase the likelihood of birds colliding with other individuals, buildings or other permanent structures^40^. Birds may suffer from disrupted and reduced periods of comfort behaviours^17^, increased air and surface pollution^41–43^, and increased vigilance at the expense of other activities such as foraging or mating^6,44,45^. Energetic costs will increase during single or multiple panic flights, with Kölzsch et al.^11^ showing a 1–10% increase in daily energy costs for multiple geese (*Anser* and *Branta* spp.) during NYE fireworks. Physiological reactions may also occur, with species experiencing increased heart rate or cortisol production^25,46^. Bögel et al.^18^ found a nearly fourfold increase in the heart rates of griffon vultures (*Gyps fulvus*) exposed to fireworks (an increase from 50 bpm (beats per minute) resting to 170 bpm during disturbance). Birds may also abandon or delay return to roost sites^11^. The downstream impacts of this should not be underestimated as they may negatively impact birds at the individual level (e.g., decrease in fitness^47^, increased mortality) and the population (e.g., through increased mortality and lower reproduction of individuals influencing population size where festivities occur during the breeding season^26,47^). Whilst we focus on the three major firework events within the UK, other, more localised events with fireworks (such as concerts) may occur during the breeding season, potentially impacting reproductive success. Whilst we acknowledge that large-scale events with fireworks are rare outside of the main event windows investigated here, the potential impact is important to consider for any organisation or individual planning such displays during the breeding period.

### Technological constraints

Radar systems overlooking large urban areas experience blockage of the signal within the radar field of view (FOV) by tall structures (such as buildings and/or pylons) or trees^48^ which diminish the integrity of the tracking algorithm. The number of tracks does not necessarily directly translate to the absolute number of individual birds in the FOV because, for example, tracks can be ‘split’. Splitting occurs when a target (i.e., a bird) is tracked by the radar for multiple detections, but then a detection is ‘missed’, if, for example, the target is obscured from the radar’s line of sight (e.g., if the bird flew behind a building). In this scenario, if the target was detected once more then this would appear as a new track. Therefore, two tracks would exist within the data for one flight from one target. One track might also represent a flock of birds if constituent birds are all flying on similar headings and at similar airspeeds. However, as the same filtering was applied to all of the data, and comparisons were made over the same durations between firework and baseline periods, flighted activity of birds should still be comparable. Indeed, no significant differences existed between the track lengths recorded during and outside firework release periods (supplementary S2). 

## Conclusions

Urban birds are often characterised as ecological generalists with traits that make them more resilient to anthropogenic disturbance, allowing them to take advantage of human-modified landscapes^19,49^. Here, however, we show that when fireworks are discharged, birds within a major UK city take to the skies in greater numbers and at higher altitudes, matching the results of a study focused on more natural habitats^21^. The results presented here add to the growing literature^11,17,21^ on wildlife disturbance from firework release whilst also being the first evidence of disturbance to birds from firework release within a city landscape. Although birds within cities are generally more resilient to disturbance than their rural counterparts, the growing intensity and the number of anthropogenic sources of disturbance, such as artificial light at night^50–52^, pollution^41,42^, and noise^6,8^, mean that fireworks cannot be viewed as a disturbance source in isolation. Instead, they add to the growing list of contributors to anthropogenic disturbance experienced by urban birds. Mitigations, such as those proposed by Stickroth^17^, including minimum distances to known roosts and water bodies, increased distances around reflecting surfaces, such as buildings and cliffs, and tighter controls on the spatial and temporal release windows of fireworks would help to reduce adverse effects of fireworks on birds. Our results also illustrate the potential of employing (L-band) radar systems for long-term monitoring of aerial bird activity within cityscapes. Such monitoring could help to track migrants and assess how activity changes above a city across extended periods and weather conditions, and during disturbances such as those shown here.

Future work should quantify the number and spatial distribution of firework discharges within urban and more natural locations to enable comparisons of disturbance to birds at the individual, population, and ecological community levels. As most fireworks within the UK are released during periods of autumnal avian migration, further work is needed to estimate the proportion of flying activity (here, the number of tracks) represented by migrants using appropriate methodologies such as moon-watching^53^, thermal cameras^54^, and acoustic recording^27^. This would allow us to assess if tighter control should be placed on firework events planned for periods of known annual avian migration fronts. Finally, combined with species-specific tracking, species-specific mitigations, such as reducing the use of fireworks in areas with high abundances of the most impacted species, could be implemented to improve the future for populations of urban birds.

## Methods

### Radar and study area

We used a Thales Aveillant 16U L-band prototype staring radar system (Fig. 1a), designed for the detection of small, low-altitude, low radar cross-section (RCS) objects, such as birds, located on the University of Birmingham’s campus (lat. 52.45°N, long. 1.92°E). This pulse Doppler radar used a broad beam on transmit and had a fully digitised 2-D receiver array that enabled it to form simultaneous multiple beams over its entire field of regard. The received pulses were integrated for roughly 0.5 s, and a threshold was applied to the radar echoes to generate up to two detections per second for a target. Multiple detections associated with a given target (i.e., a flighted object moving within the airspace generating signal above the noise floor) were linked using a real-time tracker to produce 3-D tracks that, following a classification stage, assigned labels to each tracked object. Jahangir et al.^55^ provide further details on the radar facility. The study area covered by the radar was a 90-degree azimuth sector out to a 5 km range. The area is within Birmingham, the second largest city in the UK with a population of 1.1 million people as of 2021. Birmingham contains mainly urban and suburban areas, with small pockets of grassland (parks and fields), forest, and a large body of water (Edgbaston reservoir) (Fig. 1b).

### Firework events

We selected Diwali (04/11/21), BN (05/11/21) and NYE (midnight on 31/12/21) as periods to assess how fireworks impact local airspace use by birds as these are the largest celebrations incorporating pyrotechnics within the UK. Bonfire Night is an annual event, mainly observed in Great Britain, commemorating a failed plot to blow up the Houses of Parliament in 1605. Observed on the 5th of November (and after/before depending on what day of the week the 5th of November falls in any particular year), large amounts of fireworks are discharged in publicly organised and private displays. The second celebration, Diwali, is the festival of lights, one of the major festivals celebrated by Hindus, Jains and Sikhs. The festival lasts five days during the Hindu lunisolar month Kartika, which falls between mid-October and mid-November. Here, we focus on the day of Diwali, which was 04/11/2021. New Year’s Eve is a global event where fireworks are typically set off at midnight to celebrate the start of the new calendar year. 

Celebrations with fireworks can be impacted by which day of the week they occur. If the celebration day falls on a weekday, events can be planned on the Friday evening or the weekend because they attract more attendees. Therefore, we first checked when the celebration days occurred in 2021. Diwali fell on a Thursday while BN and NYE fell on a Friday in 2021 meaning that firework events were likely to occur. To confirm that firework events occurred, we obtained data on PM_10_ (particulate matter with a diameter of 10 microns or less) concentrations (indicative of firework release^56,57^) in the area (accessed from https://sensor.community and DEFRA https://uk-air.defra.gov.uk/data/flat_files?site_id=BMLD (downloaded 09/01/23)). Sensor data indicated that pollution was high during the hours after sunset during Diwali and BN, but there was no significant increase in pollution on NYE. However, this could have been influenced by stronger winds during NYE, and an increase was observed during the evening and the day after NYE (see supplementary data Fig. S1.1). 

Based on the PM_10_ data and known firework release times (after sunset Diwali and BN and after midnight NYE), we selected a period of 18.00–22.00 h (GMT) for Diwali and BN and 00.00–00.30 h (GMT) for NYE. We then selected baseline dates of 29/10/2021–03/11/2021 for Diwali and BN, and 28/12/2021–03/01/2022 (excluding 31/12/2021) for NYE. We could not select dates after BN due to scheduled maintenance requiring the radar to be out of action. We extracted tracks across each baseline period for the exact times of day selected for the fireworks days. As a final step, we checked the local weather conditions across all the firework release and baseline periods. There was no precipitation during selected times. 

### Radar track data filtering 

We extracted all tracks from the radar for each day of the firework release and the corresponding baseline period. As a first step, we used the radar’s onboard classification system, which assigned a category (unmanned aerial vehicle (UAV, i.e., drone), aircraft, ground target, bird or unidentified) and certainty value for each detection within a track to assign an overall classification to each track (calculated as the mode classification across detections). We then used this category to remove any targets not classified as birds. As the classification system uses 30 initial detections (equivalent to 7.5 s of tracked flight) to begin classifying, any tracks below this detection threshold were removed. To filter tracks further, we calculated the mean AGH (above ground height, measured in metres above the ground (m)) and mean airspeed (calculated as a 3-D measurement in m/s) across the entirety of each track. We then removed filtered tracks that fell outside of 20–900 m (inclusive) AGH, the former being a nominal height to remove any errant tracks recorded below the building/treeline and the latter being the nominal observable altitude of the radar at its maximum range extent. Finally, we removed tracks with a mean airspeed > 30 m/s, above the maximum airspeed of bird species in the area^58,59^, but low enough to discount any tracks that may be due to fireworks (which would have had short tracks and exceptionally high speed upon detection). It is important to note here that the maximum detectable height of a target increases with the distance from the radar, but the probability of detecting a target also decreases. However, we argue that this is still a valid comparison as we are comparing the same region and 3-D space. As the tracking and classification algorithms are partly proprietary information of Thales Aveillant and beyond the scope of the current analysis, we cannot provide any further technical details about them.

### Analysis

We calculated and plotted the number of tracks in one-minute rolling windows to identify peaks visually. To assess whether the number of birds aloft and the altitude of their flights were significantly different during firework release events compared to the respective baseline periods, we applied a resampling procedure to the dataset to calculate a null distribution of mean differences (for a given measure, e.g., number of birds aloft) between the baseline and firework event periods. For example, for AGH, we calculated the actual mean difference between the average AGH of tracks during each firework period and the average AGH across tracks in the relevant baseline period. To create a null distribution, for each event, we merged the firework and baseline tracks and then randomly sampled 50% of the tracks from the combined firework and baseline data twice (once to represent the baseline period and once to represent the firework period observations) and calculated the mean difference for a given measure. This process was repeated 999 times to obtain a null distribution. We then plotted where the observed mean differences fell on the null distribution and calculated standardised effect sizes (SESs) using the formula^60^:

$${\text{SES}}\text{ = } \, \frac{\text{Observed mean difference-Average null mean difference }}{\text{SD(null mean difference)}} ,$$

with the *p*-value then calculated using a two-tailed z-test. This process was undertaken separately for the average AGH and the average minimum and maximum AGHs. We then applied the same null modelling process for the number of airborne birds within each study period. As tracks were binned into one-minute windows, we randomly sampled 30 windows for NYE and the baseline (equal to the sampled 30 min) and 240 windows for Diwali and BN (equal to the sampled 4 h). 

All analysis and plotting were conducted in R v4.1.1^61^.

## Supplementary Information

Supplementary Figures.

## Data Availability

All data and scripts have been made available to replicate the study (https://figshare.com/s/8475b4404e3c366afb11). The data are processed numbers of tracks from each period and the average track speeds and altitudes to calculate the differences in speed and altitude between the fireworks periods and their respective baselines.
