# Supplementary material for: L-band radar quantifies major disturbance of birds by fireworks in an urban area
Source: Sci Rep. 2023 Jul 26;13:12085. doi: 10.1038/s41598-023-39223-1 (PMC10372142; doi:10.1038/s41598-023-39223-1)
Supplement: Supplementary file 1 — Supplementary Figures. [file 41598_2023_39223_MOESM1_ESM.pdf]

Supplementary Material - L-band radar quantifies major disturbance of birds by fireworks in an urban area

S1 –

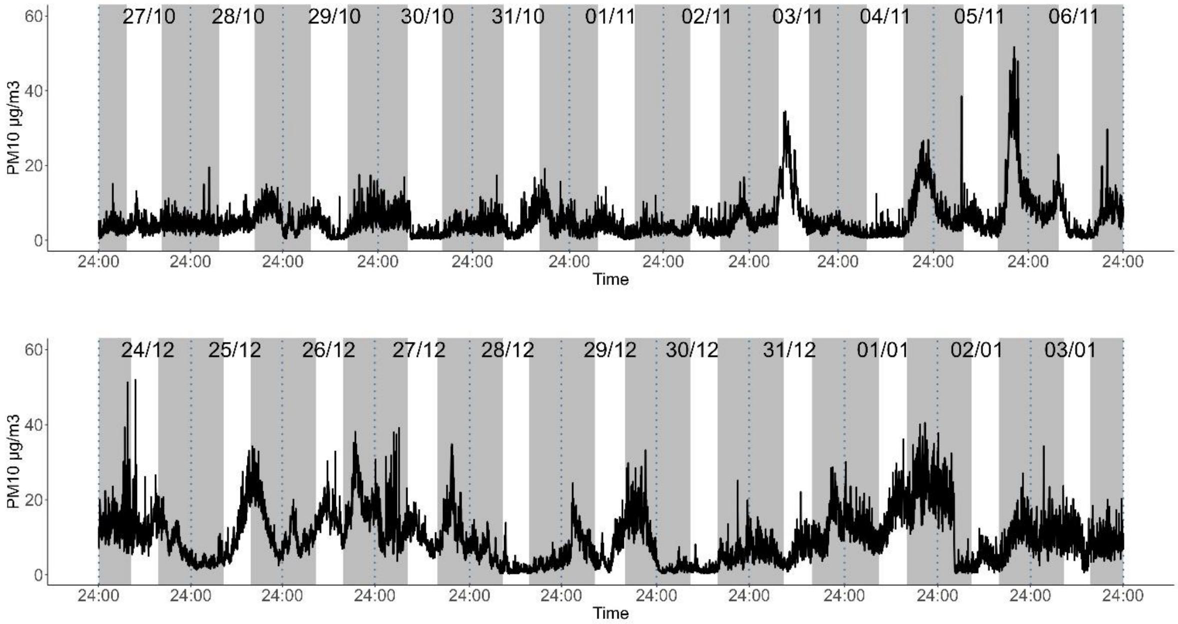

Figure. S1.1. Pollution data from a monitoring station within Birmingham, UK. The top plot displays the concentrations of PM10 before and during firework activity (Diwali (04/11) and Bonfire Night (05/11)). The bottom plot shows the same but for New Year's Eve (00:00 GMT on 31/12).

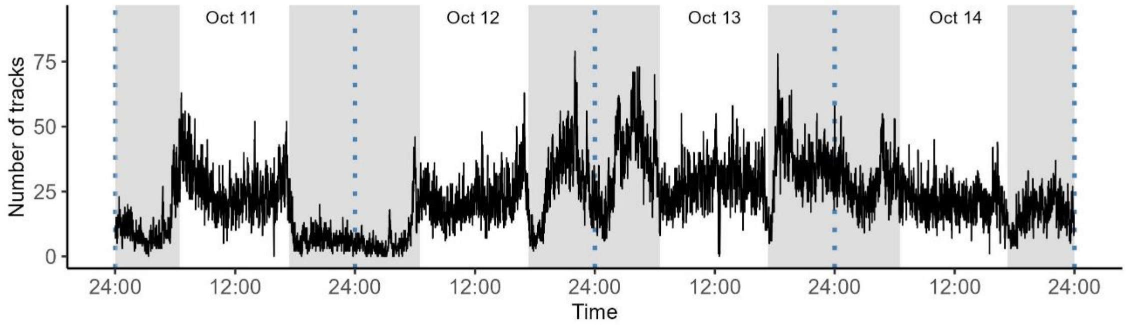

Figure. S1.2. Flighted activity recorded by radar in one minute rolling windows across a section of Birmingham, UK. Vertical dashed lines show midnight (00:00 GMT) whilst grey bars show hours between sunset and dawn.
